# A transition from unimodal to multimodal activations in four sensory modalities in humans: an electrophysiological study

**DOI:** 10.1186/1471-2202-9-116

**Published:** 2008-12-08

**Authors:** Emi Tanaka, Koji Inui, Tetsuo Kida, Takahiro Miyazaki, Yasuyuki Takeshima, Ryusuke Kakigi

**Affiliations:** 1 Department of Integrative Physiology, National Institute for Physiological Sciences, Okazaki 444-8585, Japan

## Abstract

**Background:**

To investigate the long-latency activities common to all sensory modalities, electroencephalographic responses to auditory (1000 Hz pure tone), tactile (electrical stimulation to the index finger), visual (simple figure of a star), and noxious (intra-epidermal electrical stimulation to the dorsum of the hand) stimuli were recorded from 27 scalp electrodes in 14 healthy volunteers.

**Results:**

Results of source modeling showed multimodal activations in the anterior part of the cingulate cortex (ACC) and hippocampal region (Hip). The activity in the ACC was biphasic. In all sensory modalities, the first component of ACC activity peaked 30–56 ms later than the peak of the major modality-specific activity, the second component of ACC activity peaked 117–145 ms later than the peak of the first component, and the activity in Hip peaked 43–77 ms later than the second component of ACC activity.

**Conclusion:**

The temporal sequence of activations through modality-specific and multimodal pathways was similar among all sensory modalities.

## Background

In previous studies using magnetoencephalograms (MEGs) to monitor tactile [1], auditory [2], visual [3,4] and pain [5,6] systems, we found very similar mechanisms of sensory processing among these sensory modalities. In brief, several 'early' activities appear serially with a time delay of about 4 ms at each step followed by one or two 'late' activities. In general, the 'early' activity reverses polarity twice with an interval of 10 ms, which results in a characteristic triphasic waveform, while the 'late' activity is long-lasting without a polarity reversal at such a short interval [7]. For example, following tactile stimulation, 'early' activations are elicited in area 3b, area 1 and the posterior parietal cortex in this order with a delay of 3–4 ms between each step, and then a long-lasting 'late' activity is evoked in the secondary somatosensory area. We postulate that a basic role of the 'early' activity is to receive inputs from the thalamus or convergent inputs from the thalamus and/or adjacent cortical areas and to send this information to the next point quickly, while the long-lasting 'late' activity is involved in recognition of the stimuli [2].

In the present study, we sought to compare mechanisms of sensory processing at latencies later than the 'late' activity among these sensory modalities (vision, audition, touch and pain). At first, we expected there to be unimodal and multimodal activities. Although there is a large and growing number of studies on multimodal interaction using electroencephalography (EEG) and MEG [8-13] as well as multimodal activation and interaction using functional magnetic resonance imaging (fMRI) [14-17], it is unclear whether or not the timing of the transition from unimodal to multimodal cortical activations is different among modalities. The present study manipulated the interstimulus interval (ISI) at three levels to examine the transition from unimodal and multimodal activations. In general, evoked potentials around and after 100 ms, like N1 and P3a/P3b, are more sensitively increased by increasing the ISI than earlier responses. If the difference in response amplitude between different ISIs shows the same scalp distribution among modalities, the difference in amplitude should originate from the same generator. In addition, a source analysis is helpful to estimate the location of the generator. In general, the manipulation of the ISI more strongly affects P3a and P3b, and the non-specific activities of N1, which are considered indices of orienting attention [18], compared with activities ("late" activity) within 100 ms. Because the original N1 response in any modality largely includes modality-specific activities showing a different scalp distribution depending on the modality, a comparison between the scalp distributions of the original N1 might not provide a clear-cut result. Therefore, we chose a simple manipulation of the ISI to extract more effectively the non-specific activities, to obtain a clearer result and to enable us to estimate more reliably and simply the location of the activity. Another reason for this choice is that the activities obtained by manipulating the ISI may be associated with orienting attention and later processes reflected by the non-specific N1 and P3a/P3b.

We expected the non-specific, possibly multimodal, activities obtained by manipulating the ISI are clearly found at later than 100 ms and have the same scalp distribution among modalities, but the "late" activities within 100 ms to have a different scalp distribution among modalities. The multimodal activations were expected to be in the anterior cingulate gyrus or hippocampus on the basis of a large number of previous studies performing source analyses [5,19-25] and intracranial recordings [26-29], whereas unimodal activities ("late" activities) are estimated to be in areas specific to each modality. Of final and special interest was whether or not the timing of the transition from unimodal to multimodal activations is the same among modalities.

## Methods

The experiment was performed on 14 (four females and ten males) healthy right-handed volunteers, aged 23–52 years (mean, 32 ± 8). The study was approved in advance by the Ethics Committee of the National Institute for Physiological Sciences, Okazaki, Japan, and written consent was obtained from all the subjects.

### Recordings

The EEG activity was recorded using 27 scalp electrodes placed on Fp1/Fp2, F3/F4, F7/F8, F9/F10, C3/C4, T7/T8 (T3/T4), T9/T10, P3/P4, P7/P8 (T5/T6), P9/P10, O1/O2, Fpz, Fz, Cz, Pz and Oz according to the 10–10 system. The reference electrode was placed on the nose. The impedance of the electrode was kept below 5 kΩ. The EEG signals were recorded with a bandpass filter of 0.1–100 Hz at a sampling rate of 1000 Hz, and then digitally filtered with a 70-Hz low-pass filter. The window of analysis was from 100 ms before to 500 ms after the stimulus onset, and the prestimulus period was used as the DC baseline. Four frontal electrodes (Fp1, Fp2, F9 and F10) were used for the rejection of trials containing artifacts due to blinks.

### Procedures

There were three different interstimulus interval (ISI) conditions for each modality, 0.5–0.7 s, 1.8–2.2 s and 9–11 s (2 Hz, 0.5 Hz and 0.1 Hz conditions). Therefore, there were 12 conditions (4 modalities × 3 ISIs). In each condition, 56 stimuli were presented in four separate blocks (10–17 stimuli in each block). Subjects were instructed to count the number of stimuli silently, and asked to report it in each block. If the answer was incorrect for one block out of four, the accuracy rate for the condition was 75%. In each block, the first stimulus was not included in the recording. The order of the 12 conditions was randomized among subjects.

### Stimuli

Auditory-evoked potentials (AEPs) were elicited with a 1000 Hz tone (50 ms plateau, 5 ms rise/fall) that was presented binaurally through headphones at 60 dB SPL. For somatosensory-evoked potentials (SEPs), a square wave pulse 0.5 ms in duration was delivered to the right index finger through ring electrodes with the anode and cathode at the first and second phalangeal space, respectively. The stimulus intensity was two times the sensory threshold (1.0 ± 0.4 mA). For visual-evoked potentials (VEPs), a figure of a star (white on a black background, 5.3 × 5.3° visual angle) was presented for 48 ms at the center of a screen 1.5 m in front of the subject. Noxious stimuli-evoked potentials (pain-related SEPs, pSEPs) were elicited by intra-epidermal electrical stimulation [30] using a concentric bipolar needle electrode [4] that could stimulate cutaneous A-delta fibers selectively. The electrical stimulus was a square wave pulse of 1.0 ms applied to the dorsum of the right hand between the first and second metacarpal bones. The stimulus intensity was two times the pain threshold (0.2 ± 0.1 mA). The mean visual analogue scale score (0–100) for the painful sensation was 31 ± 20.

### Analysis

Conventional averaged waveforms of the 12 conditions were obtained for each subject. Then, two difference waveforms were obtained in each modality. That is, we obtained the difference waveform between the 2 Hz and 0.5 Hz conditions by subtraction of the waveform of the 2 Hz condition from the 0.5 Hz-waveform (Sub1), and the difference waveform between the 0.5 Hz and 0.1 Hz conditions by subtraction of the 0.5 Hz-waveform from the 0.1 Hz-waveform (Sub2). Therefore, the Sub1 waveform indicated the activity that was increased in the 0.5 Hz condition as compared with the 2 Hz condition, and the Sub2 waveform indicated the activity that was increased in the 0.1 Hz condition as compared with the 0.5 Hz condition. The grand-averaged waveforms of the three conditions (2 Hz, 0.5 Hz, 0.1 Hz), Sub1 and Sub2 across all subjects were obtained and used for the analysis of topography. The averaged waveforms for the 2 Hz condition, Sub1 and Sub2 were used for source modeling.

In each waveform, the root mean square (RMS) across the 27 channels was calculated, and the field distribution was examined at several RMS peaks. The similarity of the field distribution at a certain latency point between different conditions or between different modalities was examined by determining the correlation coefficient, r [2]. In addition to the grand-averaged waveform, the similarity of the topography among modalities was also assessed using data of the 0.1 Hz condition of individual subjects.

A multi-dipole analysis was performed to separate temporally overlapping multiple sources by using the brain electric source analysis (BESA) software package (NeuroScan, Mclean, VA). Model adequacy was assessed by examining: 1) percent variance [31], 2) F-ratio (ratio of reduced chi-square values before and after adding a new source) [32] and 3) residual waveforms (that is, the difference between the recorded data and the model) as described previously [1]. Since 27 EEG channels were used in the present study, only four dipoles were allowed to be included in a model to calculate the F-ratio (the degree of freedom of a chi-square of a model is 27-6N, N = number of dipoles). This is the main reason why we used the difference waveform (Sub1 and Sub2) in this study. Our preliminary analysis showed that more than six dipoles were necessary to explain the 0.1 Hz waveform. The subtraction procedure could reduce the number of dipoles because of the presence of common source activities with similar source strength among conditions. Therefore, source modeling was applied to the 2 Hz, Sub1 and Sub2 waveforms though the goal of the analysis was to clarify the temporal sequence of each source activity in the 0.1 Hz condition, where both early and late activities were expected to be present. It is well known that the middle-long latency components of evoked potentials are sensitive to ISI (e.g. [33,34]). Only when the fourth and fifth dipoles contributed almost equally to explain the recorded data (for example, two sources in the bilateral fusiform gyrus in the 2 Hz-VEPs), was a fifth dipole included in the model. BESA uses a spherical four-shell model (the brain, cerebrospinal fluid, bone and skin). The location of each cortical source was expressed in Talairach coordinates. To confirm the reliability of results of BESA using grand-averaged waveforms, data of each subject obtained in the 0.1 Hz condition were also subjected to the source modeling. The method was identical to that for the averaged waveform. It was sometimes difficult to analyze individual data using a multi-dipole method because of the low S/N ratio. This was the main reason that we used grand-averaged data in this study. However, the evoked responses in the 0.1 Hz condition were usually enough large for this analysis at least for detecting the late activities that the present study targeted. Therefore, data of individual subjects for the 0.1 Hz condition were used for the BESA and topographical analyses, and the results were compared to those for the grand-averaged data.

## Results

### Psychophysical results

The counting task was very easy for the subjects. The mean correct answer rate was 98.2%. A two-way analysis of variance showed that the ISI (F = 3.42, P = 0.04) but not modality (F = 1.11, P = 0.35) was a significant factor modulating the correct answer rate. Bonferroni/Dunn's post hoc test indicated that the correct answer rate was significantly (P < 0.05) higher for the 0.1 Hz condition (99.4%) than the 2 Hz condition (96.4%).

### Waveform and topography

#### AEPs

**Figure 1:**
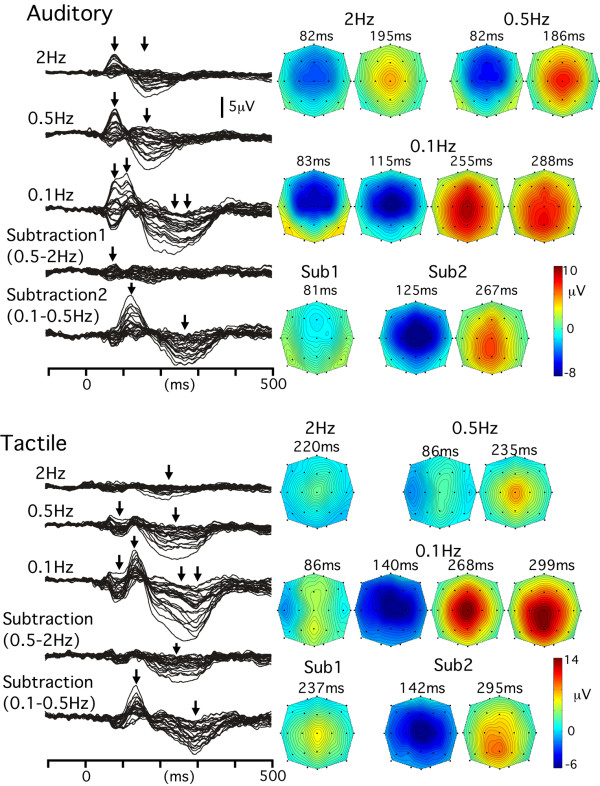
**Grand-averaged waveforms and topographies of auditory- and somatosensory-evoked potentials**. Superimposed waveforms recorded from 27 channels, obtained in the 2, 0.5 and 0.1 Hz conditions, and by the subtraction of the 2 Hz-waveform from the 0.5 Hz-waveform (Subtraction 1) and the subtraction of the 0.5 Hz-waveform from the 0.1 Hz-waveform (Subtraction 2). Isocontour maps at several peaks of the root mean square (indicated by arrows) are shown on the right.

In the waveforms of the 2 Hz and 0.5 Hz conditions, there were two RMS peaks at around 82 and 190 ms (Fig. 1). At both peaks, the field distribution was highly correlated between the two conditions (r = 0.98 and 0.99, respectively). However, the distribution was slightly different at the P7/8 and P9/10 electrodes. In the 2 Hz condition, the activity at the first RMS peak (82 ms) was negative at almost all the electrodes, but a positive activity was clearly detected at the P7/8 and P9/10 electrodes in the 0.5 Hz condition, indicating the presence of additional bilateral sources in the 0.5 Hz condition at this latency point. In confirmation of this, the Sub1 waveform showed a positivity at these electrodes at around 80 ms. In the 0.1 Hz condition, an additional negativity at around 115 ms and positivity at around 300 ms emerged. In the large positive deflection at 150–400 ms, there were two RMS peaks at 255 and 288 ms. At 255 ms, the positivity was maximal at Cz but the positive peak shifted slightly posteriorly (maximal at Pz followed by P3/4) at 288 ms, indicating that at least two distinct source activities were responsible for shaping the large positive deflection. The Sub2 waveform confirmed that there were additional large negative/positive sequential components in the 0.1 Hz condition as compared with the 0.5 Hz condition.

#### SEPs

In the 2 Hz-SEPs, a weak positivity at around 225 ms and weak activities at earlier latencies were evoked (Fig. 1). The positivity with the maximal amplitude at Cz was enhanced in the 0.5 Hz condition. In the 0.5 Hz condition, an additional negativity (maximal at T7) and concomitant positivity (Fz and Pz) at around 86 ms appeared. In the 0.1 Hz condition, a large negativity (140 ms) and positivity (200–350 ms) appeared in addition. Like waveforms of the 0.1 Hz-AEPs, the large positivity in this condition had a maximal amplitude at Cz at around 268 ms but the location of the positive peak shifted more posteriorly with an increase in latency. The field distribution pattern at the first RMS peak of the large positivity (268 ms) was correlated with that in the 0.1 Hz-AEPs (255 ms, r = 0.97 for the grand-averaged waveform and 0.9 ± 0.09 for the individual data), and that of the second RMS peak of the 0.1 Hz-SEPs (299 ms) was also highly correlated with that in the 0.1 Hz-AEPs (288 ms, r = 0.99 and 0.91 ± 0.07). Likewise, both the negativity (142 ms) and positivity (295 ms) of the Sub2-SEP waveform were significantly correlated with those of the Sub2-AEP waveform (125 and 267 ms)(r = 0.91 and 0.97, respectively), suggesting that similar cortical activities contributed to shape the waveform of the 0.1 Hz condition for AEPs and SEPs.

#### VEPs

**Figure 2:**
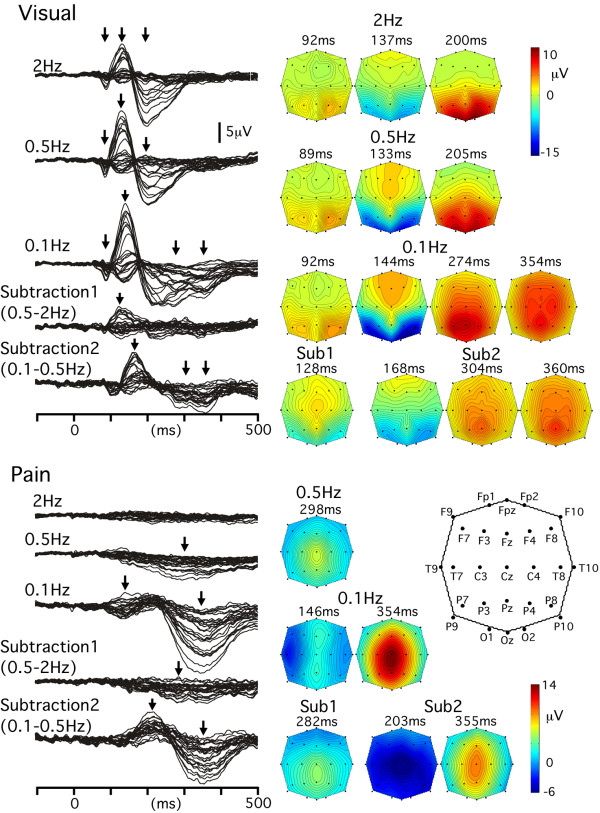
**Grand-averaged waveforms and topographies of visual- and noxious stimuli-evoked potentials**. Locations of the electrodes are shown.

Visual stimuli at 2 and 0.5 Hz evoked similar positive/negative/positive sequential components (Fig. 2). Two additional positive components peaking at 274 and 355 ms appeared in the 0.1 Hz condition. The field distribution pattern was very similar among the three ISI conditions for the first positivity peaking at around 90 ms (r = 0.92–0.98), the negativity peaking at around 140 ms (0.98–0.99) and the second positivity peaking at around 200 ms (0.97–0.98). The Sub1 waveform showed a component peaking at 128 ms, with the maximal negativity at O1 and O2 and a positivity at Cz. Similar to AEPs and SEPs, an additional negativity (168 ms) and two sequential positive components (304 and 360 ms) appeared in the 0.1 Hz condition as compared with the 0.5 Hz condition.

#### pSEPs

In the 2 Hz-pSEPs, no clear component was evoked. In the 0.5 Hz condition, a positivity peaking at 298 ms with a maximal amplitude at Cz was evoked (Fig. 2). In the 0.1 Hz condition, a large positivity peaking at Cz additionally appeared as in the other modalities. The field distribution pattern at the peak RMS of the positivity (354 ms) was similar to those of the 0.1 Hz-AEPs (288 ms, r = 0.96 for the grand-averaged waveform and 0.92 ± 0.05 for individual waveforms), 0.1 Hz-SEPs (299 ms, r = 0.98 and 0.93 ± 0.03) and 0.1 Hz-VEPs (355 ms, r = 0.91 and 0.81 ± 0.13). There was also an additional component at around 150 ms in the 0.1 Hz-pSEP as compared with the 0.5 Hz condition. At the peak RMS (146 ms), the negativity was maximal at T7 (and T8 in the ipsilateral hemisphere) and the positivity was maximal at the midline electrodes (Fz and Pz). The field distribution pattern at this latency was very similar to that at 86 ms of the 0.1 Hz-SEPs (r = 0.97 and 0.88 ± 0.07). Such a high correlation of the complicated distribution pattern between different modalities was noteworthy. The Sub2 waveform consisted of a large negativity and positivity with similar field distribution patterns in other sensory modalities (r = 0.61–0.91 for the negativity, r = 0.93–0.97 for the positivity). The relatively low correlation coefficient for the negativity was due to the concomitant existence of modality-specific activity at the latency of the negativity that was enhanced in the 0.1 Hz condition, especially in VEPs.

### Procedures of source modeling

We tried to seek the source solution responsible for the prominent component of the potentials whose topography showed high correlation among different conditions or different modalities. This suggests that there are similar generators in different conditions or different modalities. We repeated source estimation at around the peak latency of the potential component to select a robust solution having the highest GOF (or highest improvement of GOF). Once the best first source was determined, we tried to find the second source at around the peak latency of the potentials that were remained to be explained by the first source. Usually, the peak of the residual waveform was similar to that for the original waveform where the topography showed high correlation among conditions or modalities.

**Figure 3:**
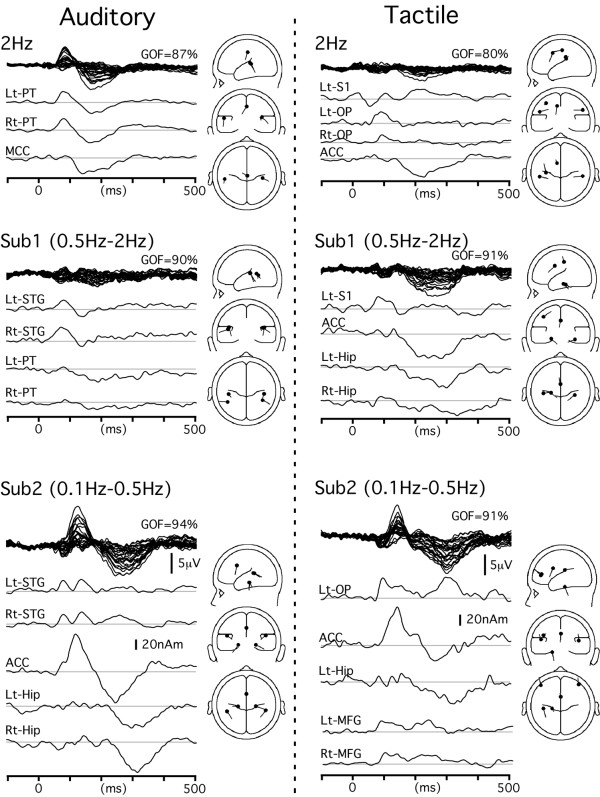
**Time course of each source activity obtained by source modeling**. Source modeling was applied to the waveform of the 2 Hz condition and two difference waveforms (0.5 Hz-2 Hz and 0.1 Hz-2 Hz). Traces show temporal profiles of source strength. Bars in the schematic drawings of the source location and orientation indicate directions of the upward deflection of the waveform. ACC, anterior cingulate cortex; MFG, middle frontal gyrus; Hip, hippocampal region; OP, opercular area; PT, planum temporale; S1, primary somatosensory cortex; STG, superior temporal gyrus.

For the waveform of the 2 Hz-AEPs (Fig. 3), we started the analysis at the first peak of RMS (82 ms). The best single source was estimated to be located in the left supratemporal plane (-55, -29, 12 in Talairach coordinates) probably corresponding to the planum temporale (PT) according to previous studies (for review, see [35]. Since the first source left substantial activity unexplained at this latency point (residual variance = 25%), we tried to find a second source at this latency. The best second source was estimated to be in the right PT (41, -27, 15), and the GOF was increased from 75 to 98% (F = 5.9, P < 0.0002) by the addition of the second source. This two-source model successfully explained the data around 82 ms, but left some activities at around 200 ms unexplained. The best source to explain the residual activity was estimated to be in the middle part of the cingulate gyrus (1, -23, 44). By adding this source, the GOF was increased from 44 to 98% (p < 0.0001). This three-dipole model provided a mean GOF value of 87% (0–500 ms), and no additional dipoles significantly improved the fit. By using similar procedures, four sources in the bilateral PT and bilateral superior temporal gyrus (STG) were estimated for the Sub1 waveform, indicating that these four source activities were stronger in the 0.5 Hz condition than the 2 Hz condition or additionally appeared in the 0.5 Hz condition. In the Sub2 waveform, bilateral STG sources were responsible for the early activity at around 90 ms. After fitting these two sources, however, large parts of the main negative/positive components were left unexplained. To explain the residual activity, the best source was estimated to be in the posterior part of the anterior cingulate cortex (ACC). This source markedly improved the fit (e.g., the GOF at 125 ms increased from 21 to 93%). However, residual activity was still clear at around 300 ms. To explain the residual activity, we had to add two more sources to the model since no single source significantly improved the fit, but residual activity was evident. The best sources were estimated to be located in the medial part of the temporal lobe in the parahippocampal gyrus of both hemispheres. After the addition of these two sources, the GOF at 300 ms increased from 64 to 98% (P < 0.02).

### Multimodal activations and modality-specific activations

**Figure 4:**
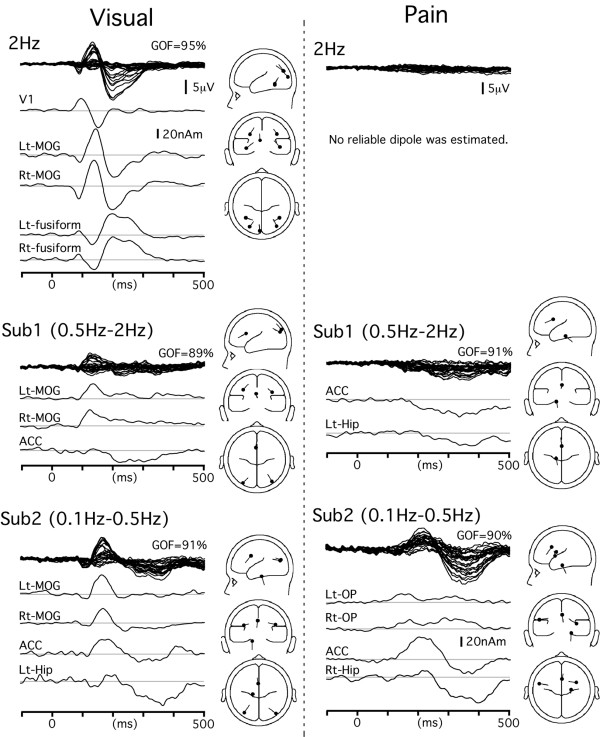
**Time course of each source activity for visual- and noxious stimuli-evoked potentials**. MOG, middle occipital gyrus.

**Figure 5:**
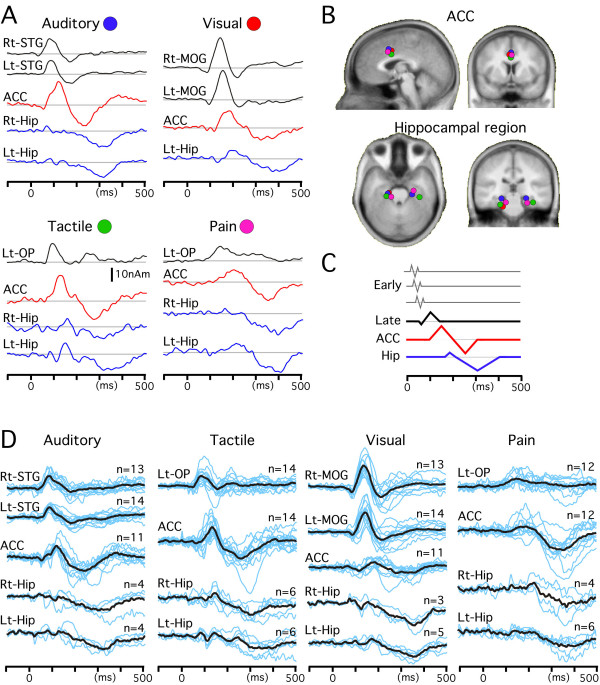
**Time course of each cortical activity in the 0.1 Hz condition**. All sources estimated in Figures 3 and 4 were applied to the waveform obtained in the 0.1 Hz condition to determine the actual time course of each activity in this condition. Among several modality-specific activities, only one or two main sources are included in this figure to clearly show the similar sequence of activations among all sensory modalities. A, temporal profiles of source strength. B, locations of the sources in the cingulate cortex and hippocampal region overlaid on the slices of the standard brain. C, schematic presentation of the common sequential activations through 'early' sensory areas, 'late' sensory areas, the ACC and the hippocampal region. D, time course of each cortical activity in the 0.1 Hz condition of individual data. Waveforms of each subject (blue lines) and their average (black) are superimposed. Note the similar temporal profile of each cortical activity between the grand-averaged data (shown in A) and individual data.

Similar procedures were applied to SEPs, VEPs and pSEPs. Figures 3 and 4 show the results of source modeling. The Talairach coordinates of each cortical source are shown in Table 1. In SEPs, VEPs and pSEPs, sources in the ACC and Hip contributed to the Sub2 waveform, like in the AEPs. That is, the ACC was the main source of activity responsible for the large negative/positive vertex potentials and the bilateral sources in Hip were also responsible for the later part of the positivity, which was consistent with similar scalp topographies of evoked potentials among modalities. The locations of the ACC and Hip sources were similar among sensory modalities (Fig. 5B).

**Table 1:** Cortical activities in response to auditory, tactile, visual and noxious stimuli.

Source	Coordinate			Peak latency (ms)	
	x	y	z	average	individual
**Auditory**					
R superior temporal gyrus	48	-35	13	81	83 ± 8
L superior temporal gyrus	-48	-38	12	87	87 ± 8
ACC	0	9	39	121/238	117 ± 13/239 ± 42
R parahippocampal gyrus	23	-21	-10	297	311 ± 25
L parahippocampal gyrus	-20	-22	-10	315	323 ± 14
					
**Tactile**					
L opercular area	54	-32	30	98	98 ± 12
ACC	0	5	32	128/272	135 ± 7/255 ± 30
R parahippocampal gyrus	35	-27	-17	315	351 ± 31
L parahippocampal gyrus	-23	-20	-14	315	315 ± 31
					
**Visual**					
R middle occipital gyrus	43	-82	16	140	144 ± 10
L middle occipital gyrus	-37	-79	15	152	146 ± 10
ACC	-2	5	37	180/314	189 ± 23/308 ± 43
L parahippocampal gyrus	-18	-21	-24	365	367 ± 10
					
**Pain**					
L opercular area	-42	-16	17	144	154 ± 21
ACC	1	11	37	200/345	210 ± 22/337 ± 25
R parahippocampal gyrus	24	-15	-18	393	386 ± 50
L parahippocampal gyrus	-13	-25	-18	403	386 ± 28

The location of each cortical source is expressed in Talairach coordinates.

The peak latency of each cortical activity for the grand-averaged data (left) and for the individual data (right, mean ± SD) is shown.

Several modality-specific activations were estimated to occur in each sensory modality. Among them, sources in the STG (AEPs), left opercular region (OP, SEPs), bilateral middle occipital gyrus (MOG, VEPs) and left OP (pSEPs) appeared to be sensitive to the ISI, that is, they were more strongly activated in longer ISI conditions.

### Time course of each source activity in the 0.1 Hz condition

Since all sources detected in the analysis described above were considered to be active in the 0.1 Hz condition, they were applied to the waveform of the 0.1 Hz condition to determine the actual time course of each cortical source activity. Figure 5A shows the source strength as a function of time of the main modality-specific source activity and multimodal activity for the waveform of the 0.1 Hz condition. The peak latency of each activity is shown in Table 1. The ACC activity was biphasic and the difference in latency between the first and second peaks was similar among modalities (117–145 ms). The activity in Hip always peaked later than the second peak of the ACC activity, and the delay (48–77 ms) was similar among modalities. In addition, the temporal sequence between the main modality-specific activation and ACC activation was similar among modalities, that is, the first component of ACC activity peaked later than the modality-specific activation by 30–56 ms. These results suggested that there were similar time courses of the multimodal activations in the ACC and Hip as well as a similar timing of the flow from the modality-specific area to the multimodal circuit among all the sensory modalities.

Results of source modeling for the 0.1 Hz waveform of individual subjects are shown in Fig. 5D. The time course of each cortical activity and the sequential pattern of activation through the sensory-specific areas, ACC and Hip were very similar to those for the grand-averaged data (Table 1). In Fig. 5D, only the main modality-specific activity and multimodal activity are shown. In addition to these sources, a significant dipole was estimated to be located in right OP (n = 5) and left S1 (n = 2) for SEP, right OP (n = 6) for pSEP, and V1 (n = 5) and the fusiform gyrus (n = 3) for VEP.

## Discussion

In our previous studies using MEG to monitor auditory [2], tactile [1], pain [5,6] and visual [3,7] systems, we showed that there were similar sequential activations through 'early' and 'late' sensory cortical areas among these sensory modalities. The results of the present study demonstrated similar time courses of activation through modality-specific areas and multimodal areas. Since the main modality-specific activations in the present study correspond to the 'late' activity in nature, these findings suggest a common temporal sequence of activations: modality-specific 'early' activity to modality-specific 'late' activity-ACC-Hip (Fig. 5C).

### Methodological considerations

In the present study, we used a three-step multiple source analysis to find cortical sources responsible for evoked potentials in the 0.1 Hz condition: 2 Hz, Sub1 (0.5 Hz-2 Hz) and Sub2 (0.1 Hz-0.5 Hz). To study the timing of sequential activations among several cortical areas, it is apparent that results would be more convincing if the subtraction procedures were not necessary. However, the present results showed that this method made it easy to find several major sources of activities responsible for evoked potentials of each ISI condition. For example, the very similar Sub2 waveforms and their topographies of all modalities clearly showed the usefulness of this method. On the other hand, however, there is a possibility that we missed minor contributors during our three-step analysis, especially weak activities that contributed to all three ISI conditions equally.

### Multimodal activation in the ACC and hippocampal region

The present results demonstrated that the main common components of the evoked potentials were the negative-positive vertex potentials that arise mainly from the ACC. It is well known that noxious stimuli evoke negative/positive vertex potentials with a scalp distribution similar to those in the present study. Source modeling of scalp potentials evoked by laser stimuli [21-23] showed that the ACC is the main generator of the vertex potentials, which was confirmed later in many studies, including intracranial recordings by Lenz et al. (1998) [36]. Several studies have demonstrated that at least some of the vertex potentials reflect sensory non-specific events [37-39]. Very similar biphasic potentials were also evoked in the ACC in response to auditory and visual stimuli in an intracranial recording study [24]. As for the negative/positive vertex potentials following tactile stimulation, the field distribution of the negative/positive potentials of the 0.1 Hz-SEPs was highly correlated with those in other modalities (e.g. r = 0.96 between SEP-N140 and AEP-N115; r = 0.99 between SEP-P299 and AEP-P288) indicating that the main generator of these potentials is not in the modality-specific area. An intracranial recording study by Allison et al. (1992) [40] supported this view by showing that the scalp negative/positive vertex potentials are not generated in the sensorimotor cortex. There have been a few studies whose results were consistent with the present findings that the main generator of the negative/positive vertex potentials in response to tactile stimuli is the ACC. An SEP study by Waberski et al. (2002) [25] reported the possible contribution of the ACC to N140, and an MEG study by Inui et al. (2003a) [5] reported the ACC activity following tactile stimulation with a peak at 128–150 ms that was coexistent with the peak (134 ms on average) of the simultaneously recorded scalp (Cz) negativity.

The cingulate cortex is an anatomically and functionally heterogeneous area, and is considered to serve cognitive, emotional, motor, nociceptive and visuospatial functions [41-44]. According to the traditional dichotomy of the cingulate cortex (anterior and posterior), the activation in the present study (BA 24/32) is located in the posterior part of the ACC. Functionally, this area in the cingulate cortex is coexistent with the cognitive subdivision of the ACC, which is activated by numerous cognitive/attentional tasks (for review, see Bush et al. 2000 [43]). The posterior part of the ACC is also a part of diffuse cortical networks sensitive to stimulus salience [17], stimulus changes [16] and oddball paradigms [20,45-47], and is thought to be the major structure in the anterior attentional system proposed by Posner and Peterson (1990) [48] and a key site in Mesulam's (1990) [49] interconnected network for directed attention. Therefore, the similar location and time course of the ACC activity among all sensory modalities in this study suggests that the ACC activity is related to modality non-specific functions. Since only a very simple counting task was used in the present study and the ACC activation was robustly obtained in the long ISI condition, the ACC activation may be related to involuntary shifts of attention to the stimulus presented against the 'silent' background [50-54]. In a classical review analyzing the component structure of auditory N1, Näätänen and Picton (1987) [18] described that the N1 consists of the modality-specific and non-specific components. One of the non-specific N1 components was predicted to be generated in the frontal lobe or deeper structure and to be associated with attentional triggering mechanisms.

Activation in the Hip has usually been studied under oddball paradigms as will be described below, and reports describing Hip activity in response to simple sensory stimuli are rare. There are several EEG and MEG studies showing Hip activity following noxious stimulation [5,23,55,56] and following tactile stimulation [5]. In these studies, the Hip activity appeared later than the modality-specific and ACC activities, which is in agreement with the present results. The important roles of the hippocampus are regarded as the memory function like the memory storage, retrieval and consolidation [57-59] or attention [60]. As described below, the Hip activity seems to be one of the major sources generating the scalp-recorded P300 (P3b), both of which we measured in this study. A series of classical psychophysical studies by Donchin and colleagues have suggested that the P3b reflects the update of context in the working memory store accompanied by the allocation of attentional resources [61]. These imply that the Hip activity we observed is associated with the memory or attentional function. It could be at least related to the more voluntary process at the later stage than the modality non-specific process that would be reflected by the ACC activity.

### Possible involvement of the ACC and Hip activities in generating the P300

In ERPs recorded under oddball paradigms, in which an infrequent target stimulus and a frequent non-target stimulus are presented in a random order, a large positive component peaking at 300 ms or more after the stimulus (P300) is elicited in response to the target stimulus [62]. The P300 component is considered to reflect fundamental cognitive processes (for reviews, see [61,63-65]). While task-relevant deviant stimuli elicit the parietocentral P300 or P3b, task-irrelevant salient stimuli inserted in the repeated target and non-target stimuli under three-stimulus paradigms [66] elicit an earlier positive deflection, the frontocentral P3a or novelty P300 [50,51,67]. The P3a has been interpreted as a neural correlate of the orienting response [64].

Both the temporal sequence and the topography of the two distinct positive components in the present study therefore resemble those of the P3a and P3b. Although the present study did not employ discrimination tasks such as those in the oddball paradigm, subjects had to count the stimuli presented against a silent background at a random ISI, and thus the 'odd' or 'infrequent' aspect of the stimulus discrimination was maintained to a certain degree and the P300 component might have been elicited. Supporting this notion, Polich et al. (1994) [68] compared the P300 elicited with auditory stimuli using a typical oddball paradigm with that elicited from a single stimulus procedure and concluded that the single-stimulus task produces the P300 in the same fashion as those elicited with the oddball paradigm. A source modeling study using ERPs by Tarkka and Stokic (1998) [69] produced similar findings.

The notion that the biphasic ACC activity in the present study might have contributed to both the negativity (fronto-central part of processing negativity or non-specific N1 component [70]; [18]) and the subsequent P3a in oddball paradigms is consistent with a recent source modeling study of scalp potentials [20], in which the main contributor to the N2/P3a components was the ACC. A source modeling study by Dien et al. (2003) [19] also found that the P3a had a source in the ACC. The hippocampal region being one of the neural origins of the P300 is consistent with previous studies using scalp potentials [69,71,72], MEG [73] and intracranial recordings [26-29]. The finding in the intracranial recording studies that the peak of the focal activity recorded from the medial temporal lobe occurred 35–100 ms later than the positive peak recorded from the scalp [28,29,74] is noteworthy, indicating that the activity in this area is mainly responsible for the late part of the P300.

### Temporal sequence of modality-specific and multimodal activations

Several modality-specific source activities were identified in each sensory modality. In general, these activities were less sensitive to the ISI than those from the ACC and Hip. However, some of these sources were activated more strongly in longer ISI conditions. These sources included STG (auditory), OP (tactile and pain) and MOG (visual). Although the significance of the sensitivity of these sources to the ISI was not clear in this study, they appear to be involved in attentional/cognitive aspects of sensory processing to a certain degree rather than the projecting function. These activities show several common features: 1) they peaked 30–56 ms earlier than the ACC source activity; 2) they lasted about 100 ms; and 3) their response latency was too long for the early (primary) activity. According to our previous findings in MEG studies, these activities correspond to the 'late' activity that follows several 'early' activities with a characteristic triphasic time course. Therefore, the present results together with our previous findings suggest a common temporal sequence of activation across all the sensory modalities: 1) the 'early' sensory cortex, 2) the 'late' sensory cortex, 3) the ACC, and then 4) the hippocampal region, each step of which roughly corresponds to 1) the quick projection of information to the next, 2) receiving, perception and integration of sensory information that has been refined at earlier stages, 3) involuntary shift of attention to perceived stimuli, and 4) voluntary aspect of cognition, memory and execution.

### Comparison with other studies on multimodal interaction

There is growing evidence for multimodal activation and interaction in humans (see review, [15,75]. Multimodal convergence has been reported in animal studies in the posterior parietal cortex, temporo-parietal junction and cingulate cortex [76-80]. It has been also reported that the cortical areas which have been considered to be exclusively modality-specific also respond to stimuli from different sensory modalities [80-84]. Most interestingly, neuronal activity can be modulated by non-auditory influences even at the primary cortical level in the primary auditory cortex [85-88]. This multimodal interaction was expected to be neural correlates of multisensory behavioral interactions observed in humans. In behavioral studies, an accelerated reaction time and an illusion of perception have been observed as a result of multisensory interaction, such as the McGurk illusion [89-91], the hearing hand illusion [92,93], and the parchment-skin illusion [94]. MEG and EEG studies have successfully demonstrated neural correlates of such multisensory interactions in humans [8-12,95-103].

The present study was planned to reveal the basic time course of unimodal and multimodal activations in a different framework from these previous studies. First of all, we attempted to know whether a similar cortical area is activated in response to sensory inputs coming individually from different modalities. Therefore, stimuli were presented individually from each modality, not the simultaneous presentation from different modalities often used in studies of multisensory interaction. In this regard, Downar et al. (2000) reported similar findings to the present study using fMRI [16,17], though the technique used and stimulus environment were different from the present ones. Then, we analyzed the difference in timing between unimodal and multimodal activations, and found a similar time course from unimodal to multimodal activations across the different modalities including vision, audition, touch and pain. This unimodal-multimodal transition expressed as a similar time course is associated with functions produced by manipulation of the ISI. Considering that the activities tested contribute to N1 and P3a/b, the unimodal-multimodal transition implies that the orienting attention and the context update are represented by electrophysiological correlates with a similar delay among those modalities tested.

## Conclusion

The present study revealed the temporal sequence of activations through modality-specific and multimodal pathways among all sensory modalities including vision, audition, touch and pain. The timing of the transition from unimodal to multimodal activations was similar among all the modalities. Take together with our previous studies investigating early cortical activities, there is thus the similar temporal sequence of activation among all sensory modalities.

## Abbreviations

ACC: anterior cingulate cortex; AEP: auditory evoked potential; BESA: brain electric source analysis; EEG: electroencephalography; ERP: event-related potential; Hip: hippocampus; ISI: interstimulus interval; MEG: magnetoencephalography; MOG: middle occipital gyrus; OP: opercular region; p-SEP: pain-SEP; RMS: root mean square; SEP: somatosensory evoked potentials; STG: superior-temporal gyrus; VEP: visual evoked potential.

## Authors' contributions

ET contributed to data collection and analysis, and drafting and revising the paper. KI contributed to planning the study, data collection and analysis, and drafting the paper. TK and TM contributed to revising the paper. YT contributed to data collection and constructing devices. RK contributed to revising the paper.
